# Depth-resolved imaging of photosensitizer in the rodent brain using fluorescence laminar optical tomography

**DOI:** 10.1117/1.JBO.25.9.096007

**Published:** 2020-09-26

**Authors:** Brandon Gaitan, Collin T. Inglut, Yi Liu, Yu Chen, Huang-Chiao Huang

**Affiliations:** a University of Maryland College Park, Fischell Department of Bioengineering, College Park, Maryland, United States; b University of Maryland College Park, College of Computer Science, College Park, Maryland, United States; c University of Massachusetts-Amherst, S617 Life Science Laboratories, Department of Biomedical Engineering, Amherst, Massachusetts, United States; d University of Maryland, Marlene and Stewart Greenebaum Comprehensive Cancer Center, Baltimore, Maryland, United States

**Keywords:** fluorescence laminar optical tomography, photosensitizer, image reconstruction, signal compensation

## Abstract

**Significance:** Previous studies have been performed to image photosensitizers in certain organs and tumors using fluorescence laminar optical tomography. Currently, no work has yet been published to quantitatively compare the signal compensation of fluorescence laminar optical tomography with two-dimensional (2-D) imaging in tissues.

**Aim:** The purpose of this study is to quantify the benefit that fluorescence laminar optical tomography holds over 2-D imaging. We compared fluorescence laminar optical tomography with maximum intensity projection imaging to simulate 2-D imaging, as this would be the most similar and stringent comparison.

**Approach:** A capillary filled with a photosensitizer was placed in a phantom and *ex vivo* rodent brains, with fluorescence laminar optical tomography and maximum intensity projection images obtained. The signal loss in the Z direction was quantified and compared to see which methodology could compensate better for signal loss caused by tissue attenuation.

**Results:** The results demonstrated that we can reconstruct a capillary filled with benzoporphyrin derivative photosensitizers faithfully in phantoms and in *ex vivo* rodent brain tissues using fluorescence laminar optical tomography. We further demonstrated that we can better compensate for signal loss when compared with maximum intensity projection imaging.

**Conclusions:** Using fluorescence laminar optical tomography (FLOT), one can compensate for signal loss in deeper parts of tissue when imaging in *ex vivo* rodent brain tissue compared with maximum intensity projection imaging.

## 1 Introduction

Since Heimstaedt and Lehmann developed the first fluorescence microscope in the early 20th century,^1^^,^^2^ it has become a critical tool for biological research. The use of fluorescent probes has allowed scientists and physicians to increase contrast and signal from a region of interest, allowing for the study of biological systems that could not previously be imaged. One of the most common uses for fluorescence is in the diagnosis, planning, and treatment of cancer. For example, fluorescence imaging has been used for guiding the resection of brain tumors. Gliolan^®^ [5-aminolevulinic acid hydrochloride (5-ALA)] is an FDA-approved imaging agent used for fluorescence-guided surgery of malignant gliomas (e.g., glioblastoma multiforme, GBM). 5-ALA is a hemoglobin precursor, with protoporphyrin IX (PpIX) being a fluorescent intermediary molecule.^3^^,^^4^ Changes in various metabolic pathways, such as the upregulation of coproporphyrinogen and downregulation of ferrochelatase, lead to the preferential accumulation of PpIX in glioma cells over healthy brain tissues.^5^ By exciting the PpIX in tumor tissues with light at wavelengths in the range of 375-nm to 420-nm during surgery, tumor margins are highlighted for better detection and resection,^6^ increasing patient mean overall survival by an average of 3 months when compared with white light techniques.^7^

Another innovation is to use fluorescence imaging to optimize photodynamic therapy (PDT). PDT is a photochemistry-based modality that involves the use of light to activate molecules called photosensitizers. The activated photosensitizers produce reactive molecular species to modulate and damage the nearby tissues.^8^^,^^9^ In addition to generating photochemistry, an excited-state photosensitizer can also emit fluorescence, which is useful for optical imaging without any additional dyes or tags.^10^ The time between the administration of the photosensitizer and the peak accumulation in the target site (e.g., tumor) is known as the photosensitizer-light interval. Previous clinical and preclinical studies have shown that having an inappropriate photosensitizer-light interval can lead to less ideal treatment outcomes.^11^^,^^12^ The amount of light to deliver to the tissue of interest is another critical parameter used to optimize PDT, and it can depend on various factors, such as photosensitizer-light interval and local photosensitizer concentration.^13^^–^^15^ Due to the fluorescent properties of photosensitizers, fluorescent-based imaging is an ideal candidate to measure these important factors.

Various imaging modalities leverage fluorescence for photosensitizer imaging. Confocal imaging is a popular modality for imaging cells and tissue in the microscopic regime. The ability to restrict the light being imaged to a thin slice allows for high resolution when imaging thicker samples due to the mitigation of out of focus fluorescence. The main drawback of this imaging modality is a small field of view ($∼1\text{-}{\mathrm{mm}}^{2}$) and a shallow penetration depth (∼50- to 200-μm).^16^^,^^17^ Widefield imaging is the most common modality used in the clinic for fluorescence imaging and is routinely used in 5-ALA-assisted fluorescence-guided resection. The advantages are apparent with this modality in that it is well studied and easy to set up, having micrometer resolution (under certain conditions), a wide field of view, and a large array of consumer-grade products to choose from. The main disadvantage comes when imaging thicker samples such as human organs, where out of focus fluorescence and attenuation caused by the tissue reduce depth penetration, resolution, and signal-to-noise ratio.^18^

Some methods have been developed to extract three-dimensional (3-D) information from brain tumor tissue. One methodology that has gained popularity in recent years is spatial frequency-domain imaging (SFDI), which was originally developed by Cuccia et al.^19^ SFDI is an imaging method in which line pairs are illuminated on the surface with at least three different frequencies, with each frequency being illuminated at various phases. The reflectance information is then used to extract scattering and absorption information of the tissue, and it has been used previously to reconstruct 3-D tomographic information.^20^ This information has been used to link variations in tissue absorption and scattering to map stromal, epithelial, and adipose tissue in breast tumor tissue.^21^ More recently, SFDI has been adapted to image photosensitizers, specifically PpIX, to locate the depth and concentration of a fluorophore by determining the optical scattering coefficient and either using a modulated light signal^22^ or two distinct emission wavelengths^23^ to extrapolate the 3-D coordinates and concentration of the photosensitizer. SFDI has been found to confer certain advantages, such as current configurations being able to image a large field of view (9-cm×9-cm) and obtain signals up to 9-mm in depth.^22^^,^^23^

Laminar optical tomography (LOT) imaging was first developed by Hillman et al. in 2004 to help overcome attenuation, compensating for signal loss when imaging an object deeper in the tissue in a similar fashion to diffuse optical tomography. Hillman et al. demonstrated LOT’s ability to image an absorptive object in a scattering medium over a $9\text{-}{\mathrm{mm}}^{2}$ area with a depth penetration of 1.2-mm.^24^ Afterward, Hillman et al. adapted the LOT system to image fluorescent signals using LOT (FLOT). Specifically, fluorescence laminar optical tomography (FLOT) was used to image the propagation dynamics of electrical waves in the heart over a $13.7\text{-}{\mathrm{mm}}^{2}$ area using voltage sensitive dyes.^25^ Chen et al. later developed an integrated system that combined FLOT with optical coherence tomography (OCT) to take advantage of FLOT’s ability to image molecular information such as fluorescence and OCT’s ability to image structural and functional information (e.g., blood flow).^26^ FLOT has also been used to measure neural activity in the brain^27^ and has even been used in photoimmunotherapy to image IR700 fluorescence distribution in a tumor at different depths to noninvasively measure therapeutic effects.^28^

Although various studies have investigated using FLOT to image fluorescence in tumors to inform treatment, no research has been carried out to image the distribution of photosensitizers in the brain using FLOT. Building off previously performed studies, the benefits of using FLOT to measure photosensitizers are clear, with the ability to compensate for signal loss caused by tissue attenuation and the ability to image in 3D. With a number of clinical studies treating brain cancer patients with different photosensitizers, including Gliolan^®^, Photofrin, and benzoporphyrin derivative (BPD) (NCT03897491, NCT03048240, NCT00870779, NCT00002647), the need for accurate photosensitizer measurements in the brain has gained greater relevancy.

In this study, we found that using FLOT to image the clinically used photosensitizer BPD, we could maintain a nonsignificant change in resolution up to 600-μm in depth. Upon comparing FLOT signal loss in the Z direction with maximal intensity projections (MIP) in phantoms, we found that two-dimensional (2-D) imaging showed an exponential decay in signal, while FLOT imaging was able to maintain up to 80% of the original signal up to 500-μm. *Ex vivo* measurements displayed similar patterns as the phantom experiment.

## 2 Methods

### 2.1 Liposome Synthesis and Characterization

BPD was incorporated into the bilayer of nanoliposomes (Nal-BPD) via freeze–thaw extrusion as described previously.^29^^–^^32^ Briefly, lipids, including dipalmitoylphosphatidylcholine, cholesterol, 1,2-distearoylphosphatidylethanolamine-methoxy polyethylene glycol and 1,2-dioleoyl-3-trimethylammonium-propane (Avanti Polar Lipids), were codissolved with BPD (50-nmoles, U.S. Pharmacopeial) at 0.15-mol. % BPD-to-lipid ratio in chloroform. A thin film, created on a rotary evaporator, was rehydrated with deionized water, subjected to freeze–thaw cycles (4°C to 45°C), and extruded through polycarbonate membranes (0.1-μm pore size) at 42°C. Unencapsulated BPD was removed by dialysis against 1× phosphate-buffered saline (PBS) overnight. The final concentration of BPD within the liposomes was determined by measuring its absorbance and using the established molar extinction coefficient in DMSO ($∼34,895\;M^{-1}\;{\mathrm{cm}}^{-1}$ at 687-nm) on a multimode microplate reader (Synergy Neo2; BioTek). The retention of BPD’s fluorescent emission was verified by the multimode microplate reader.

### 2.2 FLOT Setup

**Fig. 1:**
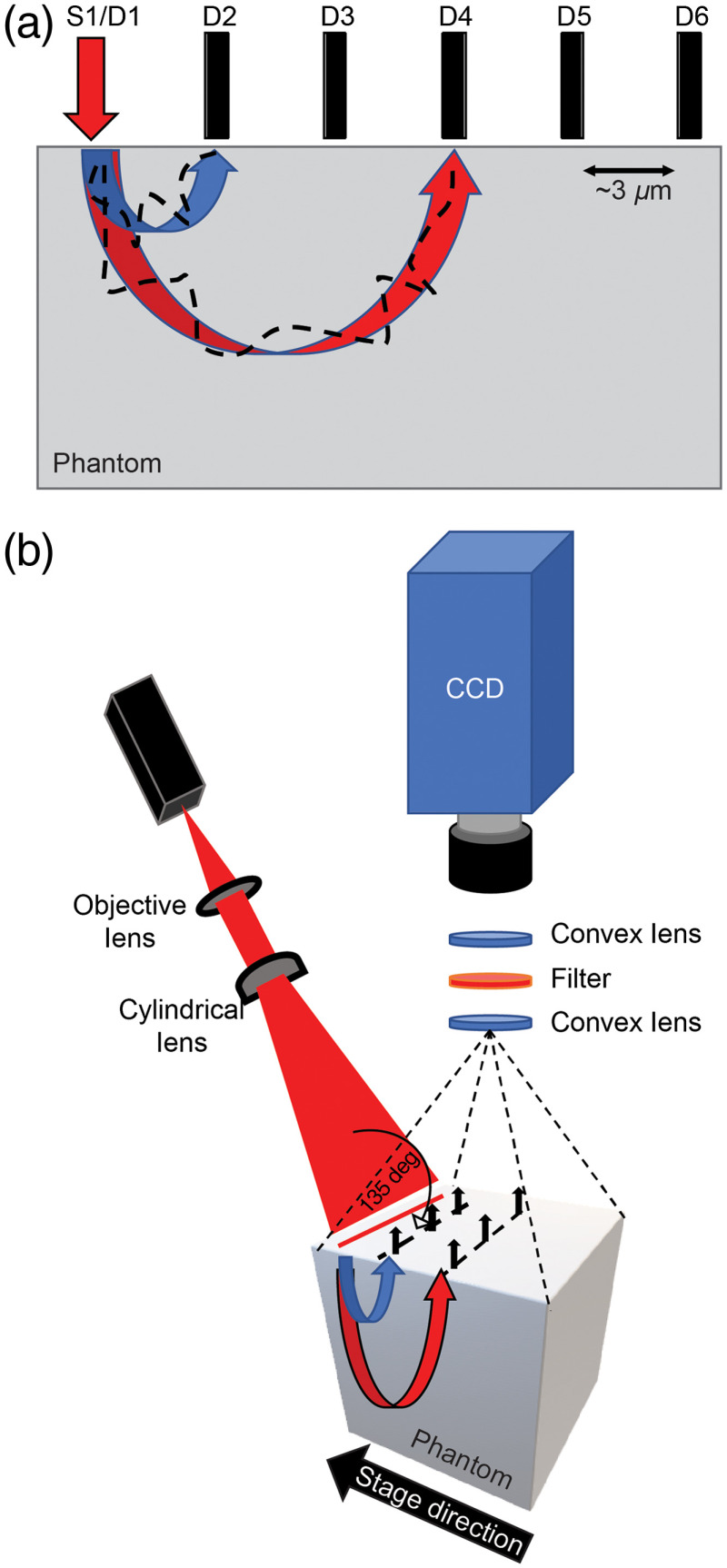
Schematic diagrams of different imaging techniques. (a) Schematic of LOT demonstrates that wider source (S1) and detector (D) offsets increase the photon migration pathway and the depth that is being imaged. (b) Schematic of the FLOT system displays a line beam (source) that allows a large region to be imaged simultaneously.

The FLOT system can be seen in Fig. 1. A 690-nm laser diode was used to excite the BPD. The laser diode was then collimated by a 50-mm lens. The collimated beam was then coupled to a cylindrical lens and focused to a line beam, with a full line-width half max of roughly 20-μm. The fluorescence was collected through an objective lens, a bandpass filter (735-nm, FF01-735/28-25, Semrock), and then a 12-bit charged coupled device (CCD) camera with a pixel size of 2.9-μm (EM-CCD, Cooke). The illumination angle was set at 135-deg on the surface of the substrate, with the CCD camera placed normal to the surface. A motorized stage was used to move the substrate perpendicular to the line illumination, taking a total of 300 images. OCT was used to provide 3-D structural information with a micrometer resolution. The *ex vivo* rat brain was first scanned with the OCT system to get 3-D images, which provided the structural information. The OCT system utilized a swept laser source (Thorlabs, Inc.), which generated a broadband spectrum of 100-nm full width at the half maximum (FWHM) centered at 1310-nm as previously described.^28^

### 2.3 FLOT Reconstruction Methodology

The FLOT system used in this study is based on the laminar tomographic imaging developed by Hillman et al.^24^^,^^25^ The design of the system is based on tomographic imaging methods such as diffuse optical tomography, taking advantage of source and detector separations, and the fact that photon scattering by structures in the brain is the dominant attenuation factor. Photons being remitted from the tissue or phantom at larger distances from the source have most likely penetrated deeper into the light scattering media, as seen in Fig. 1(a). Through the measurement of various source and detector separations, it is possible to reconstruct an entire 3-D image based solely on the fluorescence measured on the surface. As the incident light beam propagates through the tissue, some of the scattered light will be absorbed by the fluorophores of interest found in the tissue. An array of photomultiplier tubes or a CCD are then used to collect photons that are emitted by the fluorescent molecules.

Various FLOT system configurations are used by different labs. Some use point sources and detectors, using galvos to raster scan and illuminate a large area. Our FLOT system used a line field illumination, as seen in Fig. 1(b). The main advantage of this system is the simplified setup since raster scanning with galvos is not needed. The FLOT system in our lab takes a laser, which was then collimated and focused into a line beam onto the tissue. As the laser light propagated throughout the tissue, fluorescence was generated where the photosensitizer was present. The fluorescent signal was then filtered to reduce the signal caused by the excitation beam, and the remaining fluorescent signal was collected by a black and white CCD camera. Because there was always some light leakage, a line beam can be seen on the CCD camera. The observed line beam was then designated as the surface on the image. All other points on the CCD camera were then designated as detectors. This allowed us to collect many source and detector separations all at once. The tissue sample was then moved using a motorized stage at a constant velocity, allowing for different source locations on the sample.

**Fig. 2:**
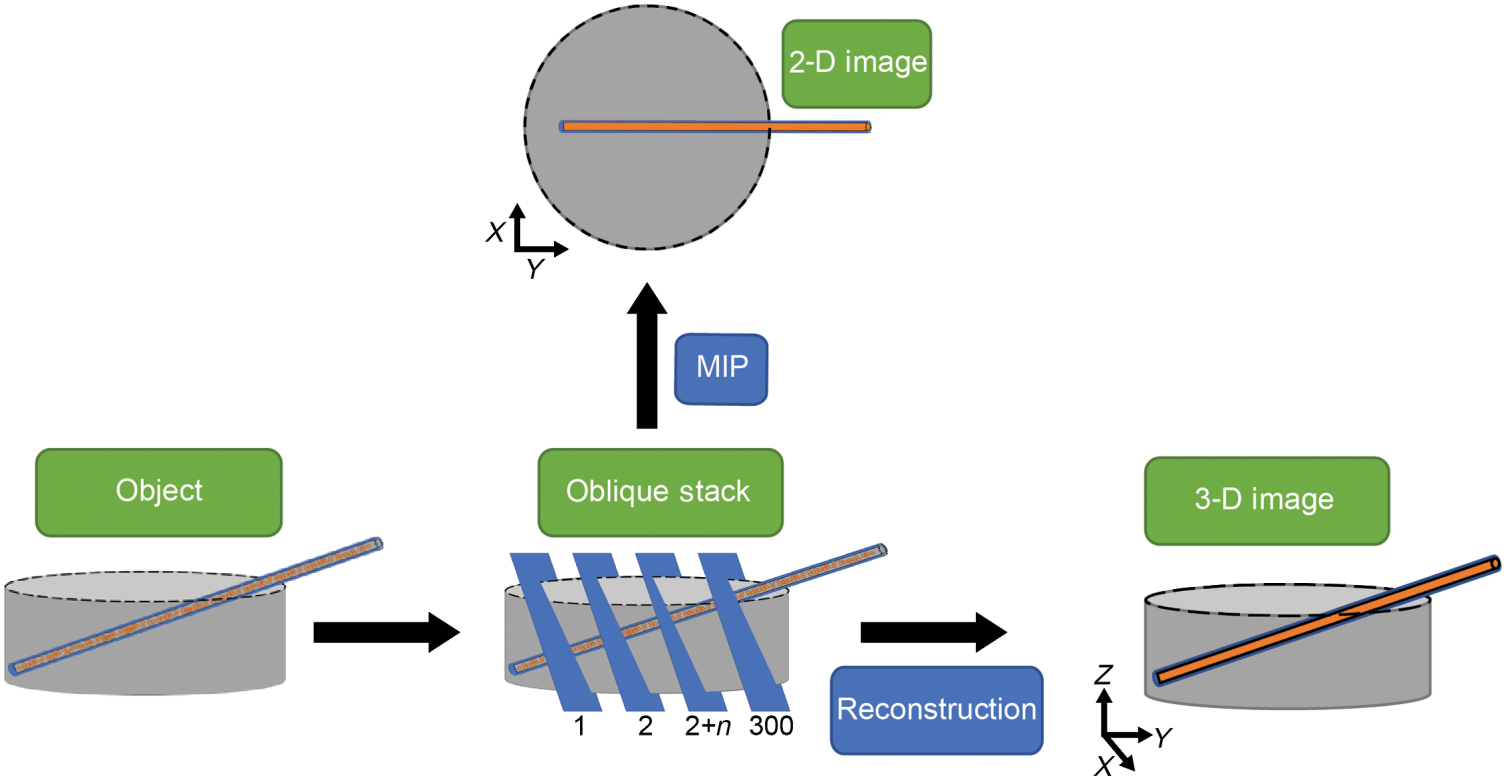
Overview of the process flow to make 2-D and 3-D images. A 2-D (MIP) image is created by imaging the object with the FLOT system and orthogonally stacking the 300 images and taking the maximal value projected in the X and Y directions. A 3-D (FLOT) image is constructed by taking the oblique stack and using it to solve the inverse problem using MCX software.

After obtaining the images, we solved the following equation, assuming the first-order born approximation

$$\Delta F(\overset{\to }{r_{s}},\overset{\to }{r_{d}})=\int G(\overset{\to }{r_{d}}-\overset{\to }{r^{\prime }})\cdot O(\overset{\to }{r^{\prime }})\cdot \Phi (\overset{\to }{r^{\prime }}-\overset{\to }{r_{s}},w)d^{3}\overset{\to }{r^{\prime }},$$ (1)

where ΔF is the fluorescence distribution in tissue and G is the probability density function that the fluorescence in position $\overset{\to }{r^{\prime }}$ will be detected by the detector in position $\overset{\to }{r_{d}}$. Φ is the distribution of the photons over $\overset{\to }{r^{\prime }}$ coming from the source position $\vec{r_{s}}$, with w being the angular momentum. O is the position of the fluorophore in the tissue.

Equation 1 can also be referred to as

$$\Delta F=W_{s.d.}\Delta O(\overset{\to }{r}),$$ (2)

where W is a weighted sensitivity matrix. After determining the parameters, we can conclude that, using the obtained ΔF and calculated $W_{s.d.}$, we can determine the actual object position in tissue [$\Delta O(\overset{\to }{r})$].

The weighting matrix can be determined by solving the radiative transfer equation. A common way to solve for the radiative transfer equation is using the diffuse approximation. This method is normally used for diffuse optical tomography due to its simplicity in implementation and because it is computationally inexpensive.^33^ The main drawback is that it is difficult for this method to resolve objects close (within scattering length) to the surface of the tissue being measured. Another method that can be used to solve the radiative transfer equation and obtain the required sensitivity matrix is using a Monte Carlo (MC) photon simulator. Although this stochastic method is more computationally intense, it provides a more accurate solution at shallower depths near the surface of the tissue. Because of this, we used an MC simulation to determine W using MCX, a software suite developed by Fang and Boas.^34^ With W estimated using an MC simulation, the inverse problem was solved using Tikhonov regularization, with the regularization parameter determined by the L-curve criterion.^35^^,^^36^

### 2.4 FLOT versus MIP

To perform the best comparison, an MIP of the oblique stack was performed on the images before reconstruction to simulate a 2-D image. MIP is a volume rendering image processing technique that is generated by projecting the volume of interest on to a viewing plane, in our case the X and Y planes. This gives us optimal contrast when imaging our phantom. A process flow for this can be seen in Fig. 2.

### 2.5 Phantom Preparation

**Fig. 3:**
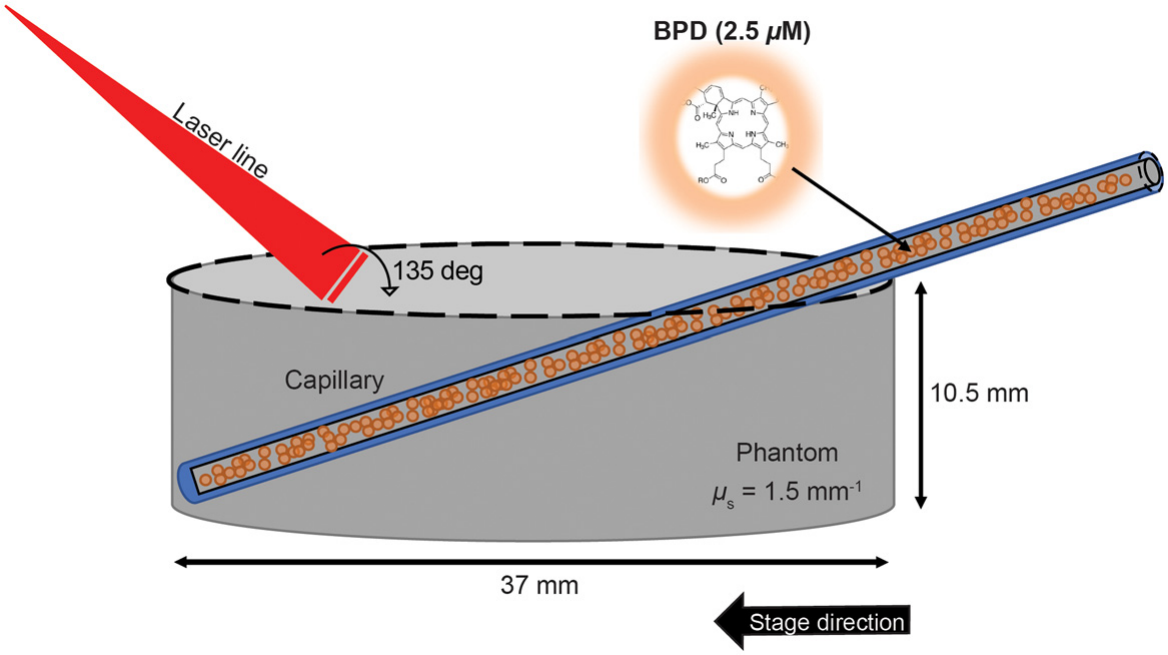
Schematic diagram of the phantom set-up. A 100-μm-diameter capillary filled with 2.5-μM of BPD is inserted into the phantom at 23.5-deg. The stationary laser line intersects the phantom at a 135-deg angle. The phantom sits upon a motorized stage that moves perpendicularly to the laser line. Not to scale.

To demonstrate the capability of the FLOT system to image photosensitizers, we filled a 100-μm glass capillary (ID: 0.10-mm, OD: 0.17-mm, VitroCom Inc.) with 2.5-μM of liposomal BPD. This capillary was then placed at a 23.5-deg angle inside a 37-mm petri dish and was sealed and fixed to the plate with an adhesive (5-Minute Epoxy, Thorlabs). Next, 2-mL of 20% intralipid solution (Sigma Aldrich Inc.) and 48-mL PBS (Sigma Aldrich Inc.) were mixed and placed into the petri dish. To determine the scattering coefficient of the phantom, we used oblique-incidence spectroscopy^37^ and found that the phantom has a scattering coefficient $\mu_{s}$ of $∼15\text{-}{\mathrm{mm}}^{-1}$ (g=0.9, n=1.33, $\mu_{a}=0.01\text{-}{\mathrm{mm}}^{-1}$, ${\mu_{s}}^{\prime }=1.5\text{-}{\mathrm{mm}}^{-1}$ at 690-nm). A schematic can be seen in Fig. 3.

### 2.6 Ex Vivo Preparation

**Fig. 4:**
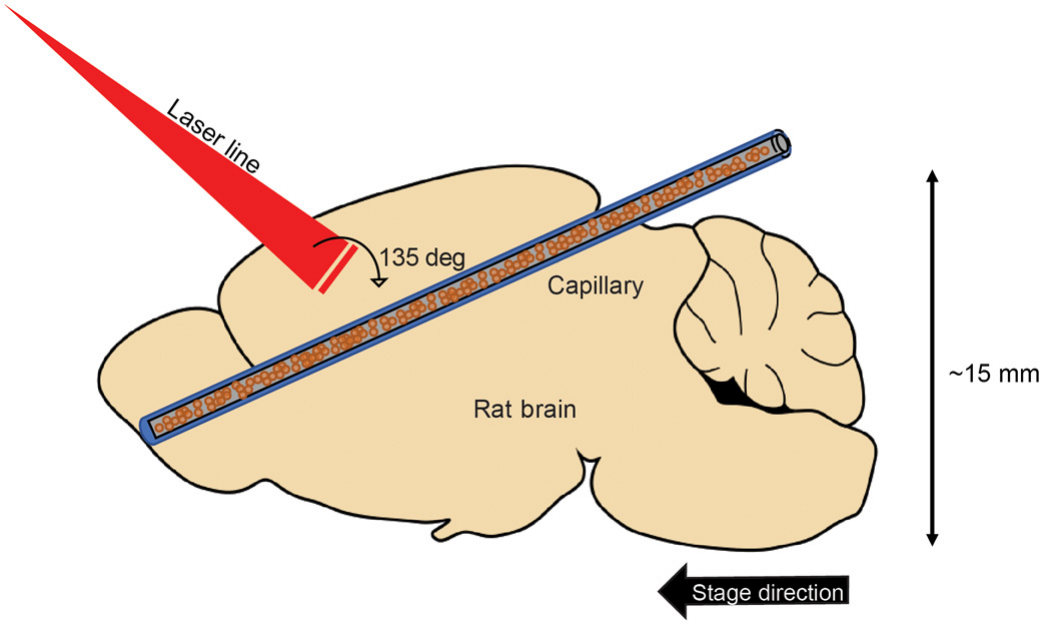
Schematic diagram of FLOT imaging the rodent brain. A 100-μm-diameter capillary filled with 2.5-μM of BPD is inserted diagonally into the rat brain. The laser line intersects the top of the brain at a 135-deg angle. The phantom sits upon a motorized stage that moves perpendicularly to the laser line. Not to scale.

The *ex vivo* portion was carried out similarly as the phantom experiment. The glass capillary (ID: 0.15-mm, OD: 0.2-mm, VitroCom Inc.) was filled with 2.5-μM of BPD and inserted into a rat brain (Innovative Research, Inc.). Rat brains were ordered snap frozen in liquid nitrogen and thawed at 4°C before being used. The brains were first imaged by the OCT system and then by the FLOT system. The images were then coregistered. A schematic can be seen in Fig. 4.

## 3 Results

### 3.1 FLOT Resolution Tests in the Lateral and Axial Direction

**Fig. 5:**
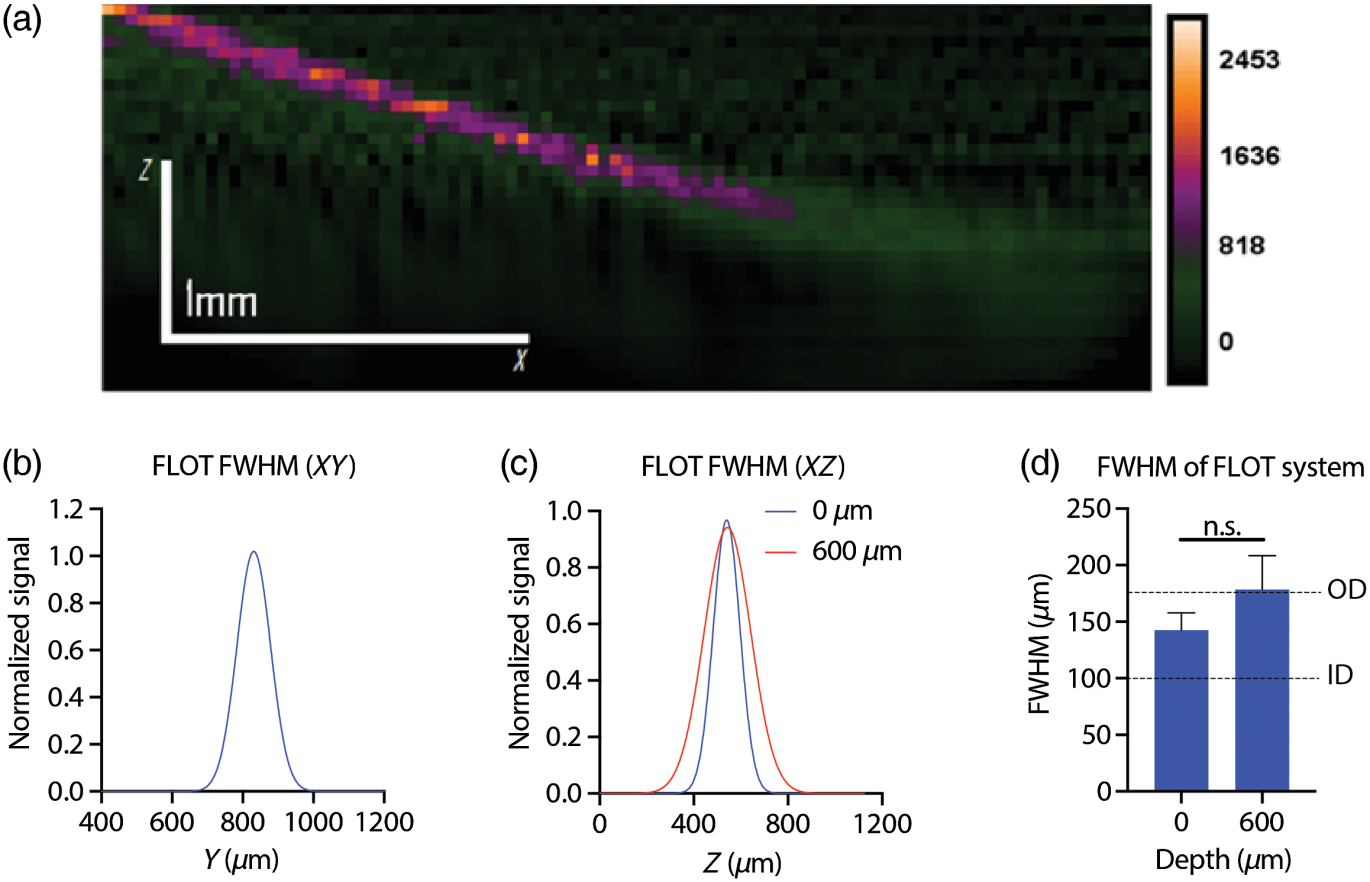
FLOT imaging of BPD within the phantom maintains a high degree of resolution at low depths. (a) XZ-image of a reconstructed FLOT image shows BPD fluorescence up to 1-mm below the surface. (b) Resolution of the FLOT system in the XY direction. (c) Resolution of the FLOT system in the XZ direction at the surface (0-μm) and 600-μm beneath the surface. (d) Comparison of XZ resolution at the surface and 600-μm beneath the surface.

Initial phantom tests demonstrate that the FLOT system can detect BPD in a high contrast environment (2.5-μM) up to a depth of 1.0 to 1.2-mm until the signal to noise drops below the limit of detection, which is defined as N+3σ, where N is the signal of the phantom with no capillary, and σ is the standard deviation of a blank image (standard deviation of noise).^38^ The shape of the capillary is preserved as it goes deeper into the phantom, demonstrating the ability to reconstruct the distribution of BPD faithfully when scanned perpendicular to the line beam. The resolution was determined by finding the FWHM of the capillary. The phantom test demonstrated a resolution of 116±5-μm in the X and Y directions [Fig. 5(b)]. To see what effect depth can have on the resolution of the system, the FWHM of the capillary was taken at different depths. The resolution at a depth of 0-μm was 143±15-μm, while at a depth of 600-μm the FWHM was 178±30-μm [Fig. 5(c)]. Although there is an increase in the resolution as we image deeper, the difference is nonsignificant up to a depth of 600-μm [Fig. 5(d)]. It must be noted that this is when the object is imaged perpendicular to the line beam, as the imaging method is not perfectly isotropic. Objects can be imaged parallel to the line beam, but this results in an expansion of the point spread function in the lateral direction.

### 3.2 MIP versus FLOT Comparison in a Phantom

**Fig. 6:**
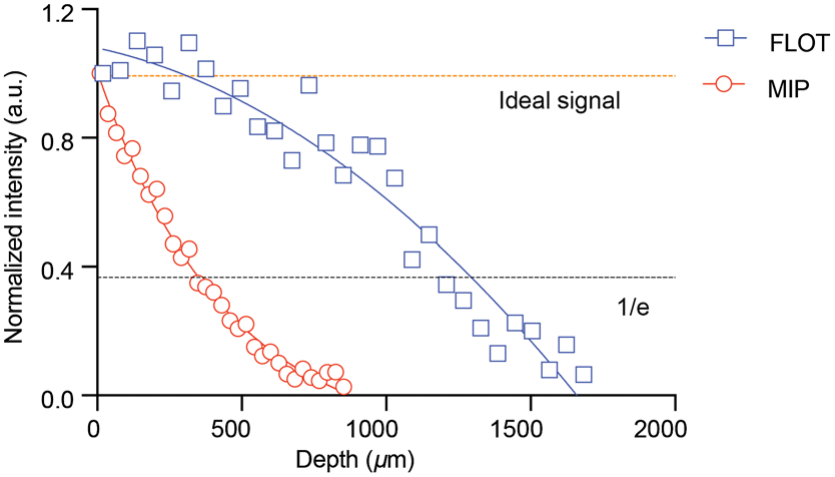
Comparison of MIP and FLOT signals at various depths in a phantom. FLOT imaging can capture BPD fluorescent signals at a twofold greater depth than MIP. The ideal signal demonstrates what the signal should appear as if the reconstruction was perfect. The 1/e line shows the point where MIP and FLOT decay to 37% of their original value.

The same phantom set up that was used to determine the resolution of the system was also used to compare signal loss in the Z direction between MIP imaging and FLOT. Both MIP and FLOT images were normalized to the fluorescent signal at Z=0-μm. Figure 6 shows the FLOT, MIP, the ideal signal, and 1/e intensity plots (e: Euler’s number). The ideal intensity is when no signal attenuation occurs when imaging deeper through a perfectly clear medium, as ideally the FLOT system would correct to this line. The 1/e line represents a signal loss of 63% (1/e), which gives a method to compare MIP signal loss with FLOT signal loss. The MIP demonstrates an exponential decay pattern versus depth, which is expected for light loss in tissue. In contrast, the FLOT system demonstrates a double polynomial decay pattern, compensating for typical signal loss caused by the phantoms attenuation. To perform a more direct comparison between FLOT and MIP imaging, the depth at which each modality reached a signal loss of 1/e was quantified. For the MIP image, the depth at which a signal of 1/e was reached was 588±27-μm, while with the FLOT system we did not see the signal decay to 1/e until 1073±118-μm beneath the surface of the phantom (p<0.05).

### 3.3 Ex Vivo Comparison of MIP and FLOT in a Rodent Brain

**Fig. 7:**
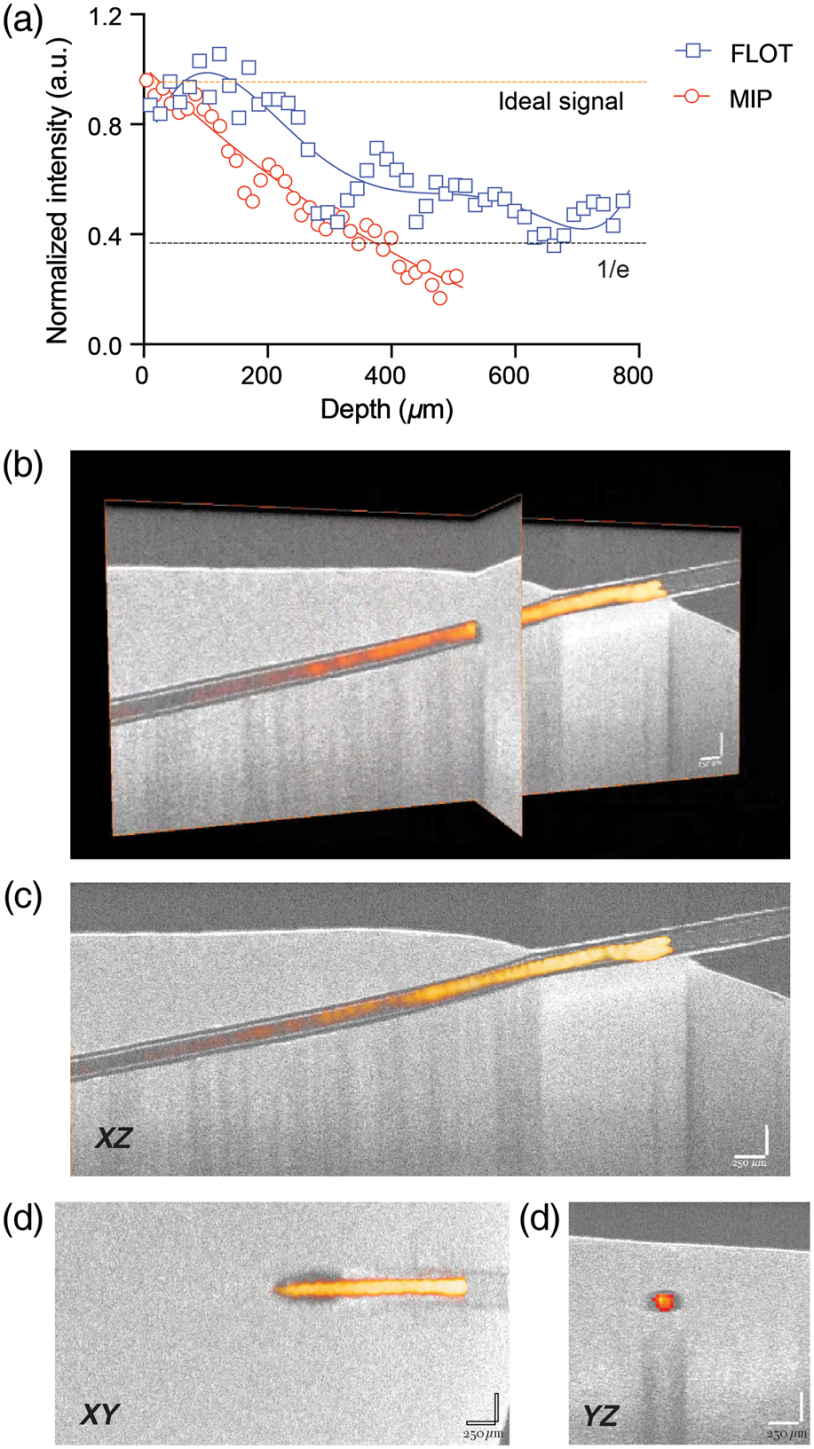
Comparison of MIP and FLOT signals at various depths in the rodent brain. (a) Normalized BPD signal captured by MIP and FLOT at various depths in an *ex vivo* rodent brain. (b) *Ex vivo* rodent brain with an FLOT capillary image (orange) coregistered with an OCT image (grayscale). (c) XZ slice of an OCT and FLOT overlay in an *ex vivo* rodent brain with a 250-μm scale bar in white. (d) XY slice of an OCT and FLOT overlay 250-μm beneath the surface of an *ex vivo* rodent brain. (e) YZ slice of an OCT and FLOT overlay 490-μm beneath the surface of an *ex vivo* rodent brain.

*Ex vivo* tests in the brain were done similarly to the phantom experiments, with a glass capillary filled with 2.5-μM BPD. A larger capillary (ID: 0.15-mm, OD: 0.2-mm) was used as the 100-μm capillary was too pliable, causing slight curvatures in the capillary and making the FLOT image more difficult to coregister with OCT. The OCT system was used to determine the ground truth for the angle of insertion of the capillary and the depth at different locations for both FLOT and MIP imaging. Decay patterns for MIP imaging demonstrate a similar decay pattern to that seen in the phantom experiments, with FLOT exhibiting signal compensation below the tissue surface as seen in Fig. 7. FLOT and OCT imaging show the ability to reconstruct the capillary faithfully. As in the phantom test, we compared the depth at which each modality reached a signal level of 37% (1/e). For the MIP image, the depth at which a signal of 1/e was reached was 334±38-μm, while with the FLOT system the signal did not decay to 1/e until 591±126-μm (p<0.05).

## 4 Discussion

Photosensitizers, such as PpIX and BPD, have been investigated for fluorescence-guided surgery and PDT. In this study, BPD was selected due to its larger maximum activation wavelength, leading to a deeper penetration depth. While PpIX has a maximal excitable wavelength of 630-nm and an emission peak at 690-nm, BPD demonstrates a maximum excitation peak at 690-nm and emission at 700-nm.^39^ BPD first gained FDA approval in the United States in 2002, specifically to treat wet age-related macular degeneration.^40^ Several studies have looked into the use of BPD in the brain, in addition to developing mathematical tools to measure the amount of reactive molecular species produced by BPD for accurate dosimetry.^41^ Our previous work demonstrated that 690-nm light-activation of BPD induces PDT effects at further depths in rat brain (1.5 to 2-cm), compared with 635-nm light activation of PpIX (0.5 to 1-cm).^39^ When imaging, we do not expect to achieve similar penetration depths when comparing the induction of PDT effects in the brain with imaging penetration, as inducing PDT effects only requires an excitation photon to reach the photosensitizer. Fluorescence imaging of a photosensitizer, on the other hand, involves several steps, including the absorption of a photon by the photosensitizer, the fluorescence to be generated (only a small fraction of the power input to the photosensitizer), the generated fluorescence to reach the surface of the tissue, and enough photons to then reach the surface to be read by a photodetector.

BPD has a low aqueous solubility (i.e., the inability to form sufficient hydrogen bonds with water), and it often requires solubilization or incorporation into a drug delivery platform for biological applications. Free-form BPD aggregates in aqueous solutions and self-quenches, resulting in inadequate bioactivity and biodistribution. Liposomal encapsulation of BPD (Visudyne^®^) allows for the retention of its fluorescent emission due to its more monomerized molecules. Although liposomal encapsulation allows for improved photosensitizer delivery compared with free-form BPD, one drawback is the heterogeneous pharmacokinetics and biodistribution of liposomes in normal tissues. The side effects of PDT on normal tissues could help potentially explain why the clinically used formulation has only modestly improved patient outcomes, as seen through numerous clinical trials (NCT00002647, NCT00049959, NCT00007969). This further demonstrates the need for an imaging system that can quantify the amount of BPD present not only at the disease site but also in fragile normal tissues, such as the brain.

In this study, we have demonstrated the capability of the FLOT system to better detect the distribution of BPD in the brain compared with MIP. The first advantage is that FLOT gives a full 3-D image up to a depth of ∼1-mm in the brain, while MIP imaging gives a depth-integrated signal of a 3-D volume that is heavily weighted toward surface readings. FLOT also helps to maintain resolution when imaging deeper in tissue. Due to scattering, the deeper the light penetrates into the tissue, the greater the light spreads,^42^ decreasing resolution. Niu et al. developed a depth compensation algorithm to improve depth localization by making a weighted matrix W, defined as

$$W={\left\{\mathrm{diag}[M(A_{l}),M(A_{l-1})\ldots M(A_{2}),M(A_{1})]\right\}}^{\gamma },$$ (3)

where M(A) is the max single value for the forward matrix from the first to the l’th layer and ϒ is an adjustable power and is varied from 0 to 3, with the value of 0 representing no use of a depth compensation algorithm.^43^ Using this algorithmic depth compensation, they found that, when measuring objects 0.6-cm in diameter separated by 1.5-cm, the object could still be resolved when imaging up to 4-cm below the surface with a ϒ of 1.3, while the objects could not be individually resolved with a ϒ of 0. These same advantages are likely the reason that we see a similar trend when comparing the resolution at the surface with that at 600-μm below the surface. We do see that the resolution decays to a greater and significant degree after this point, likely because as the depth increases the compensation that can be achieved using MCX decreases.^24^^,^^27^

The main advantage of FLOT is the signal compensation deeper in the tissue. The FLOT system without using a weighting/reconstruction method would simply be an oblique (30-deg) stack. Although this would allow for 3-D visualization, due to attenuation, the signal would be weighted toward the surface of the tissue. The MIP image demonstrates an exponential decay pattern, which has been reported through various simulations and experiments. The decay pattern in tissue is, in its most simplified form, described with the equation:

$$I(z)=I_{0}e^{-(z*\mu_{\mathrm{eff}})},$$ (4)

with z being the depth, I(z) being the intensity at the specific depth, $I_{0}$ being the intensity on the surface, and $\mu_{\mathrm{eff}}$ being the effective attenuation coefficient, where $\mu_{\mathrm{eff}}\approx \mu_{s}$ when $\mu_{s}\gg \mu_{a}$.^44^ In Figs. 6 and 7(a), an exponential decay pattern can be seen for MIP imaging, fitting prior results. The weighted matrix allows us to compensate for some of the signal lost as we image deeper into the tissue, which can be seen in Figs. 6 and 7(a). This depth compensation could be seen in similar systems, such as in Hillman’s and Tang’s previous works.^24^^,^^25^^,^^28^ Although the FLOT system does compensate for some signal loss, this compensation does deteriorate as we image deeper in the tissue. The main reason is that to reconstruct the signal, the imaging system still needs to pick up photons. Even with a perfect reconstruction method, if there are no photons collected from a specific depth, then reconstruction is impossible. This means that we are still limited by scattering as we are dependent on a photon reaching the object and the emitted photons reaching the surface.

With the system showing clear advantages over MIP imaging, there are still some improvements that could be implemented. Currently, the brain is assumed to have a uniform attenuation coefficient, but it has been demonstrated that the brain scattering coefficient varies with depth.^45^ An MC simulation that considers different layers of the brain would allow for a more faithful reconstruction. The brain also has curvatures that can increase the complexity of the boundary conditions not considered with the current iteration of the FLOT system. This can be mitigated through the integration of a mesh-based MC system and would also cut down on computational complexity.^46^

Another concern that needs to be addressed is making the FLOT system more clinically compatible. Two main issues need to be solved before the device is ready for the clinic. The first is that the FLOT system needs to be made more flexible. For example, the setup would need to be altered so that it does not depend on the sample being moved to acquire images, as moving a patient would be challenging during image-guided surgery. The second issue that would need to be solved is the coregistration of the FLOT image with conventional 2-D images. The FLOT is not meant to replace conventional imaging, but to enhance it. Overlaying FLOT with the 2-D imaging would give the surgeon the most accurate spatial information possible without having to switch between different imaging modes.

Although adaptations still have to be made to make the device clinically viable, the ability to image in full 3D and compensate for signal loss gives this system the ability to provide an image of the real distribution of photosensitizer in the brain. Another benefit compared with some other similar imaging modalities is the relatively simple hardware, as there are only five main components (lenses, laser, camera, filters, and motorized stage), and the alignment of the system is relatively simple. This makes the device likely to have a high tolerance, allowing it to be transported without worry of misalignment. Even with these adaptations, there will be some inherent limitations compared with conventional wide-field imaging that is used in the clinic. The largest limitation is the processing time necessary to make the 3-D FLOT image. Because we rely on MC simulations and reconstruction, it can take 30-s to 1-min to reconstruct an image. Because of this, FLOT is not meant to be used during the entire surgery but rather if the surgeon wants more information about the fluorophore distribution in a specific location.

Future work also needs to compare the ability of the system to measure depth and compensate for signal. A possible imaging modality to compare FLOT with is SFDI, which, as mentioned in Sec. 1, has certain configurations that use the extracted scattering and absorption coefficients to find the depth of a fluorescent inclusion in brain tissue.^22^^,^^23^^,^^47^ These systems have a similar field of view and clinical use case, so the comparison would help contrast both systems and determine which modality is best for which scenario in the clinic.

## 5 Conclusion

We investigated the 3-D distribution of BPD in phantoms and in rodent brains using FLOT imaging. The FLOT system was able to obtain images with a resolution of 110-μm in the X and Y directions and a resolution of 127 to 205-μm in the Z direction. We also compared FLOT with an MIP of oblique stacked images to perform the best possible comparison between 2-D and 3-D imaging. We found that we can compensate for signal loss deeper in both phantoms and in *ex vivo* brain tissue compared with MIP imaging. Although this method has demonstrated several advantages over MIP, several steps must be taken to make the device more clinically compatible, such as adapting the device into a more flexible form to make it easier to image the brain during surgery.
